# Actin nucleator Spire 1 is a regulator of ectoplasmic specialization in the testis

**DOI:** 10.1038/s41419-017-0201-6

**Published:** 2018-02-12

**Authors:** Qing Wen, Nan Li, Xiang Xiao, Wing-yee Lui, Darren S. Chu, Chris K. C. Wong, Qingquan Lian, Renshan Ge, Will M. Lee, Bruno Silvestrini, C. Yan Cheng

**Affiliations:** 1 The Mary M. Wohlford Laboratory for Male Contraceptive Research, Center for Biomedical Research, 1230 York Avenue, New York, NY 10065 USA; 2 Department of Reproductive Physiology, Zhejiang Academy of Medical Sciences, Hangzhou, China; 3 School of Biological Sciences, University of Hong Kong, Hong Kong, China; 4 Department of Biology, Hong Kong Baptist University, Hong Kong, China; 5 The Second Affiliated Hospital & Yuying Children’s Hospital, Wenzhou Medical University, Wenzhou, Zhejiang China; 6 S.B.M. s.r.l. Pharmaceuticals, Rome, Italy

## Abstract

Germ cell differentiation during the epithelial cycle of spermatogenesis is accompanied by extensive remodeling at the Sertoli cell–cell and Sertoli cell–spermatid interface to accommodate the transport of preleptotene spermatocytes and developing spermatids across the blood–testis barrier (BTB) and the adluminal compartment of the seminiferous epithelium, respectively. The unique cell junction in the testis is the actin-rich ectoplasmic specialization (ES) designated basal ES at the Sertoli cell–cell interface, and the apical ES at the Sertoli–spermatid interface. Since ES dynamics (i.e., disassembly, reassembly and stabilization) are supported by actin microfilaments, which rapidly converts between their bundled and unbundled/branched configuration to confer plasticity to the ES, it is logical to speculate that actin nucleation proteins play a crucial role to ES dynamics. Herein, we reported findings that Spire 1, an actin nucleator known to polymerize actins into long stretches of linear microfilaments in cells, is an important regulator of ES dynamics. Its knockdown by RNAi in Sertoli cells cultured in vitro was found to impede the Sertoli cell tight junction (TJ)-permeability barrier through changes in the organization of F-actin across Sertoli cell cytosol. Unexpectedly, Spire 1 knockdown also perturbed microtubule (MT) organization in Sertoli cells cultured in vitro. Biochemical studies using cultured Sertoli cells and specific F-actin vs. MT polymerization assays supported the notion that a transient loss of Spire 1 by RNAi disrupted Sertoli cell actin and MT polymerization and bundling activities. These findings in vitro were reproduced in studies in vivo by RNAi using Spire 1-specific siRNA duplexes to transfect testes with Polyplus in vivo-jetPEI as a transfection medium with high transfection efficiency. Spire 1 knockdown in the testis led to gross disruption of F-actin and MT organization across the seminiferous epithelium, thereby impeding the transport of spermatids and phagosomes across the epithelium and perturbing spermatogenesis. In summary, Spire 1 is an ES regulator to support germ cell development during spermatogenesis.

## Introduction

In actively migrating mammalian cells such as macrophages and fibroblasts, they generate branched (i.e., unbundled) actin filament networks and parallel actin filament bundles in lamellipodia and filopodia, respectively, by engaging two entirely different actin polymerization machineries: the Arp2/3 complex and the Spir/formin actin nucleator complex to support cell movement^1–5^. During spermatogenesis, developing germ cells, in particular post-meiotic spermatids that are nonmotile cells per se, must be transported across the entire seminiferous epithelium during spermiogenesis so that fully developed spermatids (i.e., spermatozoa) can line-up at the luminal edge of the apical compartment to prepare for their release at spermiation at stage VIII of the epithelial cycle^6–9^. While Sertoli cells are motile cells when cultured in vitro, they no longer actively migrate around the seminiferous epithelium but serve as the ‘nurse’ cells by nurturing germ cells to support their development. Furthermore, neither Sertoli nor germ cells possess lamellipodia and filopodia in vivo to support active cell movement. Instead, germ cells rely on the Sertoli cells in particular the actin- and microtubule (MT)-based cytoskeletons in Sertoli cells to provide the support and machineries so that they can be transported across the seminiferous epithelium during the epithelial cycle^10–13^. Studies have shown that the testis-specific adherens junction (AJ) known as ectoplasmic specialization (ES) that are found at the Sertoli–spermatid (step 9–18) interface (i.e., apical ES) is the only anchoring junction that supports spermatid transport during spermiogenesis; and ES is also found at the Sertoli cell-cell interface (i.e., basal ES), which is the crucial component of the blood–testis barrier (BTB) that supports preleptotene spermatocyte transport across the immunological barrier^7,8,14–16^. Since the ES in the testis is constituted and supported by an array of actin microfilament bundles and an adjacent network of MTs, it is generally accepted that the actin- and MT-based cytoskeletons in Sertoli cells play a crucial role to support germ cell transport during spermatogenesis^8,10,12,14,17,18^. Indeed, studies have shown that Sertoli cells in the testis are utilizing the Arp2/3 (actin related protein 2/3)-N-WASP (neural Wiskott-Aldrich syndrome protein) complex^19^ and formin 1^20,21^ to regulate F-actin organization at the apical and basal ES to support germ cell transport in the epithelium during the epithelial cycle. However, it remains to be investigated if Spire is expressed by Sertoli and/or germ cells and if it is involved in regulating F-actin organization in the testis.

Similar to formins (e.g., formin 1^20–22^), Spire such as Spire 1 and Spire 2 is a WH2 (WASP-homology 2, an actin monomer-binding motif consisting of ~ 17 amino-acid residues) domain-containing actin nucleator^4,23^. But, unlike formins such as formin 1 which functions as a dimerized protein, Spire is a monomeric protein capable of inducing actin polymerization via the addition of ATP-actin monomers to the filament ‘barbed end’^22^. Spire has four WH2 domains in tandem located in the center of its polypeptide sequences to recruit ATP-actin monomers to initiate actin polymerization, thus it is capable of generating long stretches of linear actin microfilaments efficiently^4,23^. These actin filaments can then be bundled via the action of actin bundling proteins Eps8^24^, palladin^25^, and plastin 3^26^ to support the actin microfilament bundles at the ES. While Spire functions as an independent actin nucleator as a monomeric protein, Spire can also dimerize when it is binding to formins, creating the formin/Spire nucleator complex to induce efficient actin filament polymerization, generating long stretches of linear actin microfilaments in mammalian cells^4,23^. Thus, in order to better understand the biology of actin polymerization that maintains actin organization at the ES, we sought to investigate the role of Spire 1 in the rat testis as reported herein.

## Materials and methods

### Animals and ethics statement

Adult male Sprague-Dawley rats (280–300 g body weight) used for various in vivo experiments, or male pups to be used at 20 days of age for the isolation of Sertoli cells for primary cultures, were purchased from Charles River Laboratories (Kingston, NY). Rats were housed at the Rockefeller University Comparative Bioscience Center (CBC) in accordance with the applicable portions of the Animal Welfare Act and the guidelines in the Department of Health and Human Services publication *Guide for the Care and Use of Laboratory Animals*. The use of animals was approved by the Rockefeller University Institutional Animal Care and Use Committee (IACUC) (Protocol Number 12–506 and 15–780-H). The use of siRNA duplexes for applicable in vitro and in vivo experiments was approved by Rockefeller University Institutional Biosafety Committee (IBC) (Approval Number 2-15-04-007). All rats were euthanized by CO_2_ asphyxiation using slow (20 ~ 30%/min) displacement of chamber air with compressed carbon dioxide in an euthanasia chamber approved by the Rockefeller University Laboratory Safety and Environmental Health (LSEH).

### Antibodies

Antibodies used for various experiments reported here were obtained commercially from different vendors except otherwise specified. The RRID (Resource Identification Initiative) numbers of all antibodies are included in Table 1, as well as their working dilutions used for various experiments.

**Table 1:** Antibodies used for different experiments in this study

Antibody (RRID No.)	Host Species	Vendor	Catalog Number	Working Dilution	
				IB	IF/IHC
Spire1(AB_11156859)	Rabbit	Abcam	ab13043	1:1000	1.100
Spire1(AB_10960492)	Rabbit	Sigma-Aldrich	HPA040942	—	1.100
Spire1 (AB_2286545)	Rabbit	Santa Cruz Biotechnology	sc-85162	—	1.100
ZO-1(AB_2533938)	Rabbit	Invitrogen	61–7300	1:250	1.100
Occludin(AB_2533977)	Rabbit	Invitrogen	71–1500	1:250	1.100
CAR(AB_2087557)	Rabbit	Santa Cruz Biotechnology	sc-15405	1:200	1:50
JAM-A(AB_2533241)	Rabbit	Invitrogen	36–1700	1:250	—
Claudin 11(AB_2533259)	Rabbit	Invitrogen	36–4500	1:250	—
N-cadherin(AB_2313779)	Mouse	Invitrogen	33–3900	—	1:100
N-cadherin(AB_647794)	Rabbit	Santa Cruz Biotechnology	sc-7939	1:200	—
β-catenin(AB_2533982)	Rabbit	Invitrogen	71–2700	—	1:100
β-catenin(AB_634603)	Rabbit	Santa Cruz Biotechnology	sc-7199	1:250	—
Vinculin(AB_477629)	Mouse	Sigma-Aldrich	V9131	1:250	—
Espin(AB_399174)	Mouse	BD Biosciences	611656	1:250	—
Filamin A(AB_2106406)	Rabbit	Santa Cruz Biotechnology	sc-28284	1:200	—
Arp3(AB_476749)	Mouse	Sigma-Aldrich	A5979	1:3000	1:100
Eps8(AB_397544)	Mouse	Invitrogen	610143	1:5000	1:100
Plastin 3(AB_2636816)	Rabbit	Abcam	ab137585	1:500	—
Palladin(AB_2158782)	Rabbit	Proteintech Group	10853-1-AP	1:1000	1:100
Formin 1(AB_2105244)	Mouse	Abcam	ab68058	1:500	1:50
N-WASP(AB_2288632)	Rabbit	Santa Cruz Biotechnology	sc-20770	1:200	—
Fascin(AB_627580)	Mouse	Santa Cruz Biotechnology	sc-21743	1:200	—
Ezrin(AB_304261)	Mouse	Abcam	ab4069	1:300	—
β1-integrin(AB_2130101)	Rabbit	Santa Cruz Biotechnology	sc-8978	1:200	1:100
α-tubulin(AB_2241126)	Mouse	Abcam	ab7291	1:1000	1.200
Detyrosinated α-tubulin (AB_869990)	Rabbit	Abcam	ab48389	1:200	
β-tubulin(AB_2210370)	Rabbit	Abcam	ab6046	—	1.200
β-actin(AB_630836)	Goat	Santa Cruz Biotechnology	sc-1616	1:200	—
				—	
Vimentin(AB_628437)	Mouse	Santa Cruz Biotechnology	sc-6260	1:200	—
GAPDH(AB_2107448)	Mouse	Abcam	ab8245	1:1000	—
EB1(AB_397891)	Mouse	BD Biosciences	610534	1:200	
Laminin γ3(AB_2636817)	Rabbit	Cheng Lab^a^	—	1:200	
Goat IgG-HRP(AB_634811)	Bovine	Santa Cruz Biotechnology	sc-2350	—	1:3000
				—	
Rabbit IgG-HRP(AB_634837)	Bovine	Santa Cruz Biotechnology	sc-2370	—	1:3000
				—	
Mouse IgG-HRP(AB_634824)	Bovine	Santa Cruz Biotechnology	sc-2371	—	1:3000
				—	
Rabbit IgG-Alexa Fluor 488 (AB_2576217)	Goat	Invitrogen	A-11034	1:250	
Rabbit IgG-Alexa Fluor 555 (AB_2535850)	Goat	Invitrogen	A-21429	1:250	
Mouse IgG-Alexa Fluor 488 (AB_2534088)	Goat	Invitrogen	A-11029	1:250	
Biotinylated Mouse IgG(AB_2313581)	Horse	Vector Laboratories	BA-2000	—	1:300

Abcam, Cambridge, MA; Sigma-Aldrich, St. Louis, MO; Santa Cruz Biotechnology, Santa Cruz, CA; Invitrogen, Life Technologies, Carlsbad, CA; BD Biosciences, San Jose, CA; Proteintech Group, Chicago, IL. *IB* immunoblotting, *IF* immunofluorescence, *RRID* Resource Identification Initiative. Antibodies used herein cross-reacted with the corresponding proteins in rats

^a^ Yan HH, Cheng CY (2006) Laminin alpha 3 forms a complex with beta3 and gamma3 chains that serves as the ligand for alpha 6-beta1-integrin at the apical ectoplasmic specialization in adult rat testes. J Biol Chem 281: 17286–17303

### Isolation and primary culture of Sertoli cells

Sertoli cells were isolated using 20-day-old rat testes as described^27^. Freshly isolated Sertoli cells were seeded on Matrigel- (BD Biosciences, San Jose, CA)-coated culture dishes (either 6, 12- or 24-wells), coverslips (placed in 12-well dishes) and bicameral units (Millipore, Billerica, MA) (placed in 24-well dishes) at a density 0.4–0.5, 0.03–0.04, and 1.0 × 10^6^ cells/cm^2^ respectively, in serum-free DMEM/F-12 (Gibco) medium, supplemented with growth factors and gentamicin in a humidified atmosphere of 95% air/5% CO_2_ (vol/vol) at 35 °C^27^. For 6-well and 12-well dishes, each well contained 5 or 3 ml F12/DMEM medium for specific biochemical assays or for immunoblottings. But for IF, each well (with coverslip) contained 2 ml F12/DMEM. For bicameral units placed in 24-well dishes, the apical and the basal chamber contained 0.5 ml F12/DMEM. All media were supplemented with growth factors and gentamicin. On day 2 (about 36 h after cell plating), residual germ cells were removed by a brief hypotonic treatment (20 mM Tris, pH 7.4 at 22 °C, 2.5 min) as described^28^ so that these cultures with ~98% pure with some cellular debris and without detectable contaminations of either germ cells, Leydig cells and/or peritubular myoid cells using corresponding primer pairs and/or antibodies against specific marker proteins for RT-PCR or immunoblottings as described^29^. These Sertoli cell cultures were used for experiments on day 3 with an established functional tight junction (TJ)-permeability barrier, and ultrastructures of TJ, basal ES, gap junction, and desmosome that mimicked the Sertoli cell blood–testis barrier (BTB) in vivo were also detected as earlier described^30–32^.

### Knockdown (KD) of *Spire 1* by RNA interference (RNAi) in Sertoli cells in vitro

*Spire 1* was silenced in Sertoli cells by RNAi to assess the effects of its KD on Sertoli cell function. In brief, Sertoli cells cultured alone on day 3 with an established functional TJ-permeability barrier were transfected with *Spire 1*-specific small interfering RNA (siRNA) duplexes vs. non-targeting negative control (Dharmacon) duplexes at 100–150 nM (for IB and actin polymerization/bundling assay) or 50–100 nM (for IF) using RNAiMAX (Life Technologies) as a transfection reagent for 24 h as earlier described^26^ (Table 2). Thereafter, cells were washed thrice to remove transfection reagents, replaced with fresh F12/DMEM and cultured for an additional 24 h before RNA was extracted for analysis by RT-PCR or qPCR (day 5). For IF, IB or actin polymerization/bundling assay, cells were cultured until day 6 before termination. For cultures to be used for IF, cells were co-transfected with 1 nM siGLO red transfection indicator (Dharmacon) to track successful transfection. In each experiment, replicates or triplicates were used for each treatment vs. control groups, and each experiment was repeated with *n* = 3 using different batches of Sertoli cells.

**Table 2:** siRNA duplexes used for RNAi experiments

Gene	Product	Cat. No.	Target sequences (5′–3′)	Targeted Region
*Spire-1*	ON-TARGETplus rat *spire1* (307348) siRNA-SMARTpool	L-092190-02	CAGACUAGAUGUUACUACAAUUAAGACUUUGUGGACGUCUUAACAUCUCAAACGAAAUGAUGAGGGAUUUGCGAAA	ORF3′UTRORFORF
*Non-Targeting Control*	ON-TARGETplus Non-targeting Pool siRNA duplexes	D-001810-10	UGGUUUACAUGUCGACUAAUGGUUUACAUGUUGUGUGAUGGUUUACAUGUUUUCUGAUGGUUUACAUGUUUUCCUA	––––

ORF, open reading frame; UTR, untranslated region

### Knockdown of *Spire 1* in adult rat testes in vivo

*Spire 1* was silenced in adult rat (~280–300 g body weight) testes in vivo by transfecting testes with *Spire 1* siRNA duplexes vs. non-targeting control using Polyplus in vivo-jetPEI (Polyplus-transfection S.A., Illkirch, France) as a transfection reagent according to manufacturer’s instructions as earlier described^33,34^, which was shown to have a considerable improvement in transfection efficiency (at ~ 70%) when compared to conventional transfection medium (at ~ 30%)^34–36^. In brief, siRNA duplexes (500 nM) and siGLO red transfection indicator (Dharmacon) (40 nM) were constituted in 100 µl of transfection solution containing 1.7 µl in vivo-jetPEI (an adult rat testis was at ~1.6 *g* in weight, with a volume of ~ 1.6 ml) according to manufacturer’s recommendations with N/P = 8 (note: N/P ratio refers to number of nitrogen (N) residues of jetPEI/nucleotide phosphate (P), wherein the jetPEI concentration is expressed in nitrogen residues molarity and 1 µg of plasmid DNA contains 3 nmol of anionic phosphate; i.e., 500 nM (11.09 µg) siRNA required 1.7 µl in vivo-jetPEI), and administered to each testis using a 28-gauge 13 mm needle attached to a 0.5 ml insulin syringe. The needle was inserted from the apical to the basal end of the testis vertically in which the right testis was transfected with the *Spire 1* siRNA duplexes vs. the left testes transfected with the negative non-targeting control siRNA duplexes. As the needle was withdrawn apically, transfection solution was released and gradually filled the entire testis to avoid an acute rise in intra-testicular hydrostatic pressure. Transfection was performed on day 1, 2 and 3 (triple transfections, *n* = 2 rats) and in some experiments, transfection was performed on day 1, 3 and 5 (triple transfections, *n* = 3 rats). Rats were euthanized on day 5 (*n* = 2 rats) or day 7 (*n* = 3 rats), respectively. Testes were removed immediately, frozen in liquid nitrogen or fixed in Bouin’s fluid or modified Davidson’s fixative^37,38^ for their subsequent use. Since the phenotypes in these two groups of rats were similar, data from both sets of experiments were pooled for analysis with *n* = 5 rats.

### Assessment of Sertoli cell TJ-permeability barrier in vitro

Sertoli cells cultured in vitro assembled an intact epithelium with a functional TJ-barrier, capable of resisting the conductivity of electrical current delivered by a Millipore Millicell-ERS (electrical resistance system) meter that was sent across the barrier. This resistance was then recorded by quantifying the transepithelial electrical resistance (TER) in ohms (Ω) by placing two electrodes across the Sertoli cell epithelium, one in the apical and one in the basal compartment of the bicameral units. In brief, Sertoli cells were plated on Matrigel-coated Bicameral units (diameter 12 mm; pore size 0.45 µm, effective surface area 0.6 cm^2^; EMD Millipore) at 1.0 × 10^6^ cells/cm^2^. Each bicameral units was placed inside the well of a 24-well dish with 0.5 ml F12/DMEM each in the apical and the basal compartments. *Spire 1* was KD in Sertoli cells cultured alone on day 3 using specific siRNA vs. non-targeting negative control duplexes at 150 nM for 16 h, and Sertoli cell TJ-permeability barrier function was monitored daily by quantifying TER across the cell epithelium as described^27^. In each experiment, each treatment and the control group had triplicate or quadruple bicameral units. Each experiment was done with *n* = 3 using different batches of Sertoli cells, excluding pilot experiments to assess the optimal concentrations of siRNA duplexes.

### BTB integrity assay in vivo

The Sertoli cell BTB integrity in vivo was assessed by the ability of the Sertoli cell TJ/basal ES-barrier to block the diffusion of a membrane impermeable biotinylation reagent EZ-Link Sulfo-NHS-LC-biotin (Thermo Fisher Scientific, Waltham, MA) across the barrier as earlier described^35^. In brief, rats under anesthesia using ketamine HCl (60 mg kg b.w., i.m.) with xylazine as an analgesic (10 mg/kg b.w., i.m.) received 100 µl of 10 mg/ml EZ-Link Sulfo-NHS-LC-biotin by loading the biotin under the tunica albuginea with a 29-gauge needle of *n* = 3 rats for each group including control groups. About 30 min thereafter, rats were euthanized by CO_2_ asphyxiation, testes were immediately removed, freezed in liquid nitrogen. Thereafter, frozen 10 µm-thick cross-sections were obtained in a cryostat at −22 °C, fixed in 4% PFA in PBS for 10 min and incubated with Alexa Fluor 488-streptavidin (1:250) for 30 min. Microscopic slides were mounted with Prolong Gold Antifade reagent with DAPI (Life Technologies). Testes from normal (untreated) rats served as negative control and rats received 3 mg/kg b.w. cadmium chloride (i.p.) (for 3 days) served as a positive control which is known to induce irreversible BTB damage^39^. Semi-quantitative data to assess the BTB integrity were obtained by quantifying the distance (D) of the biotin (D_Biotin_) traversed from the basement membrane vs. the radius of a seminiferous tubule (D_Radius_). For oblique sections of the tubules, D_Radius_ was obtained using the average of the shortest and the longest distance from the basement membrane. About 50 randomly selected tubules from each testis of *n* = 3 rats with a total of 150 tubules were examined (including the control groups).

### RNA extraction, qPCR and RT-PCR

Total RNA was isolated from rat testes, Sertoli cells, germ cells and brain using Trizol reagent (Life Technologies) as described^26,40^. 2 µg total RNA was reverse transcribed by Moloney murine leukemia virus reverse transcriptase (M-MLV) (Promega, Madison, WI) according to manufacturer’s instructions to obtain cDNAs which served as templates for subsequent PCR. To quantify *Spire 1* or *Spire 2* steady-state mRNA level, PCR was performed using cDNA products obtained above as templates with a primer pair specific for *Spire 1* or *Spire 2* vs. *S16* (Table 3). Authenticity of PCR products was verified by gel electrophoresis, which was then extracted for direct DNA sequencing at Genewiz (South Plain field, NJ). Each RT-PCR experiment was performed with *n* = 3 using different batches of Sertoli cells or germ cells vs. rat testis and brain from 3 different male rats. qPCR was performed as described^34^, the mRNA level of *Spire 1* and *Spire 2* was analyzed by QuantStudio^TM^ 12 K Flex Real-Time PCR System (Thermo Fisher, Waltham, MA) with Power SYBR Green Master Mix (Applied Biosystems, Foster City, CA) according to the manufacturer’s instructions (*n* = 3). *Gapdh* was used as an internal control for normalization. The specificity of the fluorescence signal was verified by both melting curve analysis and gel electrophoresis. The expression level of the target gene was determined using 2^-ΔΔC^_T_ method.

### Protein lysate preparation, protein estimation and immunoblot (IB) analysis

Cells or testes were lysed in immunoprecipitation (IP) lysis buffer (50 mM Tris, containing 0.15 M NaCl, 1% Nonidet P-40 (vol/vol), 1 mM EGTA, 10% glycerol (vol/vol), pH 7.4 at 22 °C, freshly supplemented with protease inhibitor mixture (Sigma-Aldrich) and phosphatase inhibitor cocktail II (Sigma-Aldrich)), to be followed by an 8-s sonication (2 × , with 1 min interval in ice) using a Cole-Parmer Ultrasonic Processor (Model CPX130PB, Cole-Parmer, Chicago, IL) to obtain protein lysates by placing microfuge tubes on ice. For testes samples, we routinely used a sample:IP lysis buffer of 1:5. After centrifugation at 16,000 ×* g* for 1 h at 4 °C, clear supernatant was used for subsequent experiments. Protein estimation was performed using a Bio-Rad DC protein assay kit (Bio-Rad Laboratories, Hercules, CA) with BSA as a standard. Immunoblot analysis was performed as described^34^ using equal amount of total protein lysate between samples in each experiment: 20 or 60 μg protein for cell or testis lysates, respectively, using corresponding antibodies for different marker proteins (Table 1). Chemiluminescence was performed using an in-house prepared kit as described^41^, and signals were detected using an ImageQuant LAS 4000 (GE Healthcare Life Sciences) Imaging system and ImageQuant software (Version 1.3). GAPDH and β-actin served as a protein loading control. Protein band intensities were evaluated by ImageJ 1.45s which was obtained at http://rsbweb.nih.gov/ij (National Institutes of Health (NIH), Bethesda, MD). All samples within an experimental group were processed simultaneously to avoid inter-experimental variations. Each sample had triplcates in both silencing and control groups with *n* = 3 independent experiments using different batches of Sertoli cells or testes from three different rats.

**Table 3:** Primer pairs used for RT- and qPCR to assess the steady-state mRNA level of target genes

Gene	GenBank accession number	Primer pairs	Nucleotide position	Amplified PCR product (bp)
*Spire1*	NM_001107381.1	Forward, 5′-GCAGCTCATCGACCAAAT-3′Reverse, 5′-AAACAGTGCCCGACATAC-3′	394–411593–610	217
*Spire2*	NM_001127538.1	Forward, 5′-AGCCCGTGGGTTTGGTTCTC-3′Reverse, 5′-TTCCTCAGGCGTGCTCATCTC-3′	1107–11261267–1287	181
*S16*	NM_001169146.1	Forward, 5′-TCCGCTGCAGTCCGTTCAAGTCTT-3′Reverse, 5′-GCCAAACTTCTTGGATTCGCAGCG-3′	87–110448–471	385

### Immunofluorescence analysis (IF), F-actin staining and fluorescence image analysis

IF was performed using frozen cross sections of testes at 7 μm obtained in a cryostat at −22 °C, or fresh Sertoli cells cultured on coverslips as earlier described^26,42^. Sections or cells were fixed in 4% paraformaldehyde (PFA) or ice-cold methanol for 10 min (min), permeabilized in 0.1% Triton X-100 for approximately 5–10 min, blocked in 10% goat serum (v/v) or 5% BSA (w/v) in PBS (10 mM sodium phosphate, 0.15 M NaCl. pH 7.4 at 22 °C). Thereafter, samples were incubated with a specific primary and the corresponding secondary antibodies (Table 1), and co-stained with 4′,6-diamidino-2-phenylindole (DAPI) (Sigma) to visualize cell nuclei. Slides were mounted in Prolong Gold Antifade reagent (Invitrogen, Life Technologies). For F-actin staining, testis sections or Sertoli cells were incubated with Alexa Fluor 488 phalloidin (Invitrogen) according to manufacturer’s instructions. Images were examined and acquired using a Nikon Eclipse 90i Fluorescence Microscope system equipped with Nikon Ds-Qi1Mc or DsFi1 digital camera and Nikon NIS Elements AR 3.2 software (Nikon, Tokyo, Japan) and saved in TIFF format. Image overlays were performed using Adobe Photoshop CS4 (San Jose, CA). Fluorescence intensity was analyzed by ImageJ 1.45 s (NIH, Bethesda, MD) or Nikon NIS Elements AR (Version 3.2) software package. Sections or cells in an experiment including both control and treatment groups were analyzed in a single experimental session to eliminate inter-experimental variations. Data shown were representative micrographs from a single experiment, and each experiment had three coverslips of cells or different cross-sections of testes. Each experiment was repeated at least three times using different batches of Sertoli cells or testes from 3 rats which yielded similar results. For fluorescence intensity or distribution analysis in Sertoli cells or seminiferous tubules of testes, at least 200 cells or 200 cross-sections of tubules were randomly selected and examined in both experimental and control groups in an experiment, and a total *n* = 3 experiments were performed.

### Assessing the ratio of F (filamentous) actin to G (globular) actin by a spin-down assay

The relative ratio of F:G actin in Sertoli cell or testis lysates following *Spire 1* silencing was assessed according to the manufacturer’s instructions (Cat No. BK037, Cytoskeleton, Denver, CO) with minor modifications. In brief, Sertoli cells (~ 500 µg protein from a single well of a 6-well dish at 0.4 × 10^6^ cells/cm^2^) or testes (~ 1 mg) were homogenized in F-actin stabilization buffer, pre-cleared by centrifugation at 350 *g* for 5 min at room temperature to remove cell debris, followed by centrifugation at 100,000 *g* at 37 °C for 1 h to separate F-actin from G-actin. Supernatant (~2 ml, containing G-actin) was collected, pellet (containing F-actin) was re-suspended in 300 µl 8 M urea. Thereafter, 60 µl of supernatant and 60 µl of pellet of each sample were analyzed by immunoblotting for β-actin. Phalloidin (0.1 µM, actin stabilizing agent) vs. urea (80 mM, actin depolymerization agent) was used as the corresponding positive and negative controls. This thus assessed any changes in the relative ratio of F-actin incorporated into the cytoskeleton vs. the G-actin pool in the cytosol in Sertoli cells (or testes) following *Spire 1* knockdown when compared to testes in control group transfected with non-targeting negative control siRNA duplexes.

### Actin bundling assay

Actin bundling assay was performed as earlier described^20,26^ to assess the ability of Sertoli cell lysates to bundle premade F-actin (purified and polymerized F-actin from human platelets) in vitro following *Spire 1* silencing compared to the corresponding controls according to the manufacturer’s instructions (Cat No. BK013, Cytoskeleton). In brief, Sertoli cell lysates (obtained by centrifugation following cell or tissue lysis in a Tris-based lysis buffer^20,26^) in 10 µl (containing ~ 20–40 µg total protein) obtained from cells following Spire 1 kD vs. control cells were incubated with 40 µl of F-actin (at 21 µM) for 1 h at room temperature to allow bundling of F-actin, to be followed by centrifugation at 14,000 ×* g* at 24 °C for 5 min to pellet bundled F-actin from the linear unbundled F-actin and G-actin in the supernatant. Pellet was dissolved in 30 µl sterile H_2_O and 2 µl supernatant of each sample were analyzed by immunoblotting using an anti-β-actin antibody. All samples, including both *Spire 1* KD vs. controls were analyzed in a single experiment to avoid inter-experimental variations.

### Actin polymerization assay

Actin polymerization assay was performed as described^20,26^ to assess the ability of Sertoli cell lysate following *Spire 1* silencing vs. controls to polymerize pyrene actin oligomers in vitro according to manufacturer’s instructions (Cat No. BK003, Cytoskeleton). In brief, Sertoli cell lysate (obtained by centrifugation following cell or tissue lysis in a Tris-based lysis buffer^20,26^) in 30 µl (containing approximately 60–120 µg total protein) was incubated with 60 µl of pyrene actin oligomers at 0.4 mg/ml. Polymerization was initiated by adding 10 µl of a 10× actin polymerization buffer to each sample in a Corning 96-well black flat bottom polystyrene microplate (Corning, Lowell, MA, USA). Fluorescence kinetics were monitored from the top in a FilterMax F5 Multi-Mode Microplate Reader and the Multi-Mode Analysis Software 3.4 (Molecular Devices, Sunnyvale, CA) at room temperature (fluorimeter settings, measurement type: kinetic, 100 cycle, 20 s interval; excitation wavelength: 360 nm; emission wavelength: 430 nm; integration time: 0.25 ms). Actin polymerization rate assessed by fluorescence intensity increase rate was obtained by linear regression analysis using Microsoft Excel 2016 (Microsoft, WA). Phalloidin (1 µM, an actin stabilizing agent) vs. urea (100 mM, an actin depolymerization agent) recommended by the manufacturer was used as the corresponding positive and negative controls. For all the actin assays, the same amount of Sertoli cell lysate (40 µg total protein) or testis lysate (60 µg total protein) from each sample was also analyzed by immunoblot to confirm the knockdown of *Spire 1*. Each sample had replicates and each experiment was repeated at least 3 times (i.e., *n* = 3) using different batches of Sertoli cells or testes from *n* = 3 rats which yielded similar results.

### Microtubule spin-down assay

MT spin-down assay was performed as earlier described^21,43^. This assay estimated the relative level of polymerized MTs vs. free tubulins in Sertoli cell cytosol according to the manufacturer’s protocols (Cat No. BK038, Cytoskeleton, Denver, CO). In brief, Sertoli cells were homogenized in 37 °C pre-warmed lysis and MT stabilization buffer (100 mM PIPES, 5 mM MgCl_2_, 1 mM EGTA, 0.1% NP-40, 0.1% Triton X-100, 0.1% Tween 20, 0.1% 2-mercaptoethanol, 30% glycerol, pH 6.9) with a 25-gauge syringe needle, pre-cleared by centrifugation at 2000 ×* g* for 5 min at 37 °C to remove cellular debris, followed by centrifugation at 100,000 ×* g* at 37 °C for 30 min to separate polymerized tubulins/MTs (pellet) from tubulin monomers (supernatant). The supernatant was collected, and the pellet was re-suspended in 250 µl 2 mM CaCl_2_. Cell lysates, pellet and supernatant were then used for IB using an anti-β-tubulin antibody. Paclitaxel (20 µM, also known as Taxol, a MT stabilizing agent) vs. CaCl_2_ (2 mM, a MT depolymerization agent) was used suggested by the manufacturer to serve as the corresponding positive and negative controls by including either paclitaxel or CaCl_2_ in the Sertoli cell lysate when the polymerized tubules/MT and free tubulin monomers were obtained as described above. In short, this assay assessed changes in the relative distribution of MTs/polymerized tubulins vs. free/non-polymerized tubulin monomers in Sertoli cell cytosol following *Spire1* knockdown when compared to control cells transfected with non-targeting negative control siRNA duplexes.

### Histological analysis

Testes obtained from rats after euthanasia by carbon dioxide asphyxiation were immediately fixed in Bouin’s fixative (Polysciences) or modified Davidson’s fixative^37,38^, and then embedded in paraffin. Sections at 5 µm thickness were obtained with a microtome, mounted on microscopic slides and staining with hematoxylin and eosin after deparaffinization. For histological analysis, at least 5000 tubules in each sample (grouped as stages I–V, VI–VIII, XI–XIV) were randomly selected and examined using cross-sections of a testis for a treatment vs. control group, and representative findings reported herein were derived from separate experiments with *n* = 4 rats which yielded similar results. Analysis was focused on stage VI–VIII and XI–XIV tubules because defects in spermatid or phagosome transports were notably detected at these stages of the epithelial cycle.

### Statistical analysis

In each experiment, the treatment and the control groups had 2–4 replicates or at least 4 rats (and for each time points wherever applicable), and all experiments were repeated at least 3 times using different batches of Sertoli cells or rats. Data analyses were performed using SPSS 16.0 (SPSS, IL). The bar and line graphs were plotted with OriginPro 8 (OriginLab, MA) or Excel 2016 (Microsoft, WA, USA). Data are presented as the mean ± s.d. of *n* = 3 to 5 experiments (or n = 4 rats). Data were subjected to homogeneity test for variance, analyzed by Student’s *t*-test (2-tailed), and *P* value < 0.05 was considered statistically significant.

## Results

### Expression of Spire 1 in Sertoli and germ cells in rat testes and its distribution in the seminiferous epithelium during the epithelial cycle

**Fig. 1:**
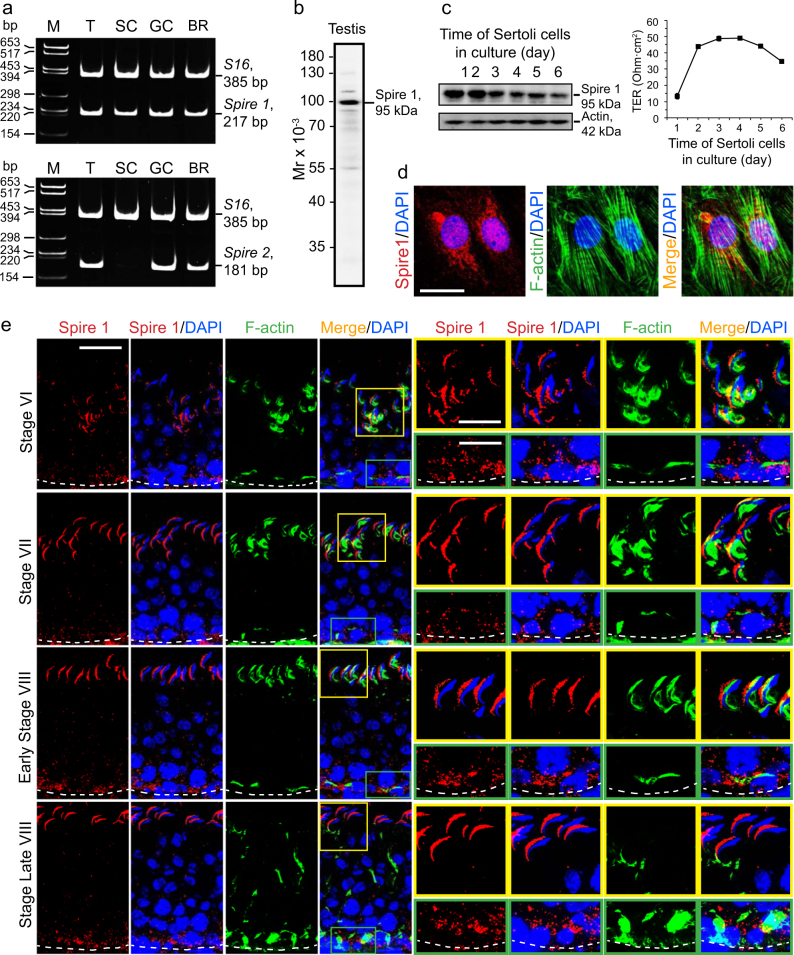
Expression, and cellular and stage-specific localization of Spire1 in the rat testis. **a** A study by RT-PCR using corresponding primer pairs specific to different target genes (Table 3) was performed to illustrate the expression of *Spire 1* in adult rat testes (T), Sertoli cells (SC), and germ cells (GC) vs. brain (BR; positive control) with *S16* served as a loading control. *Spire 2* was also expressed in T, GC and BR, but not SC under the conditions used in our experiments. M, DNA markers in base pairs (bp). The identity of the PCR products was confirmed by direct nucleotide sequencing. **b** Specificity of the anti-Spire 1 antibody (Table 1) was assessed by immunoblotting using lysates of adult rat testes (T) at 80 µg protein. **c** Changes in the relative steady-state protein level of Spire1 expressed by Sertoli cells (left panel) were detected during the assembly of the Sertoli cell TJ-permeability barrier (right panel) when cultured in vitro for 6 days in serum-free F12/DMEM using Sertoli cell lysates (40 µg protein per lane) with β-actin as a protein loading control. Assembly of the TJ-barrier was assessed by a rise of transepithelial electrical resistance (TER) across the Sertoli cell epithelium which blocked the conductivity of current across the barrier. A considerable rise of actin nucleation protein Spire 1 expression was noted during the initial phase of TJ-barrier assembly which became plateau when the barrier was established by ~day 3. **d** Staining of Sertoli cells with anti-Spire1 antibody (red fluorescence) to illustrate cellular localization of Spire 1 which was shown to partially co-localize with F-actin (visualized by Alexa Fluor 488 phalloidin, green fluorescence) in Sertoli cell cytosol. Sertoli cell nuclei were visualized by DAPI (blue). Scale bar, 40 µm. **e** Stage-specific expression of Spire1 (red fluorescence) and its co-localization with F-actin (green fluorescence) in the seminiferous epithelium of adult rat testes. Cell nuclei were visualized by DAPI (blue). Spire1, mostly expressed at the convex (dorsal) side of spermatid heads, was found to partially co-localize with F-actin at the apical ES (yellow boxes) and also at the basal ES/BTB (green boxes) near the basement membrane (annotated by dashed white line). Scale bar, 80 µm; inset, 40 µm

**Fig. 2:**
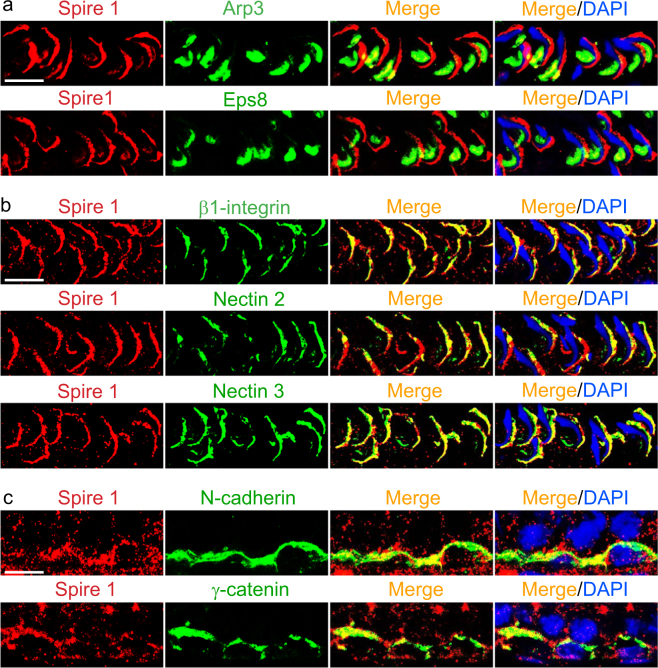
Spire1 is a component of apical and basal ES in the seminiferous epithelium of adult rat testes. **a** Dual-labeled immunofluorescence (IF) analysis of Spire1 (red fluorescence) with two ES regulatory proteins Arp3 (green fluorescence) and Eps8 (green fluorescence) at stage VII to early VIII tubules. Since Arp3 and Eps8 were mostly expressed at the concave side of spermatid heads as bulb-like structures, only partial localization with Spire 1 were detected because Spire 1 only weakly expressed at this site but robustly expressed at the convex side of spermatid heads. Scale bar, 20 µm, applies to other micrographs. **b** Dual-labeled IF analysis showed almost superimposable co-localization of Spire 1 (red fluorescence) with apical ES proteins (green fluorescence) β1-integrin (Sertoli cell-specific), nectin 2 (expressed by both Sertoli cells and spermatids) and nectin 3 (elongated spermatid-specific) in stage VII to early VIII tubules since these apical ES proteins also expressed predominantly at the convex side of spermatid heads. Scale bar, 20 µm, applies to other micrographs. **c** Dual-labeled IF analysis illustrated partial co-localization of Spire 1 (red fluorescence) with basal ES/BTB proteins (green fluorescence) N-cadherin and γ-catenin at the BTB site. Scale bar, 20 µm, applies to other micrographs

A study by RT-PCR using a primer pair specific to Spire 1 (Table 3) has shown that the actin nucleation protein Spire 1, but not Spire 2, was expressed in Sertoli and germ cells in the testis (Fig. 1a). Using an antibody specific to Spire 1 (Fig. 1b), the expression of Spire 1 by Sertoli cells when cultured in vitro to assemble the tight junction (TJ)-permeability barrier (Fig. 1c) rose steadily but remained plateaued when the barrier was established by day approximately 2–3 (Fig. 1c). Expression of Spire 1 was detected across the Sertoli cell cytosol, partially co-localized with actin microfilaments (Fig. 1d). Spire 1 was expressed in the seminiferous epithelium, prominently associated with apical ES at the Sertoli-elongating/elongated spermatid interface such as in stages VI–VIII tubules and conspicuously found at the convex (dorsal) side of spermatid heads, co-localizing with F-actin; it also expressed in the basal compartment near the basal ES/BTB and partially co-localized with F-actin (Fig. 1e). As noted in stage VII tubules when Spire 1 expression was high (Fig. 1e), dual-labeled immunofluorescence analysis (IF) in higher magnified images (Fig. 2a) had shown that Spire 1 was prominently expressed at the convex side of spermatid heads, but some minor but consistent expression was also detected at the concave (ventral) side, co-localizing with Arp3 (a branched actin nucleation/polymerization protein known to convert linear actin microfilaments to a branched actin network) and Eps8 (an actin barbed end capping and bundling proteins) at the concave side of spermatid heads (Fig. 2a) where Arp3 and Eps8 were robustly expressed in stage VII tubules, consistent with earlier reports^19,24^. Also, Spire 1 co-localized with apical ES proteins β1-integrin (expressed by Sertoli cells at the apical ES^44,45^), nectin 2 (expressed by both Sertoli cells and spermatids^46^) and nectin 3 (expressed by elongating/elongated spermatids^47^ at the apical ES) since these three apical ES proteins all expressed prominently at the convex side of spermatid heads (Fig. 2b). At higher magnification, Spire 1 was prominently expressed at the basal ES/BTB, co-localizing with basal ES proteins N-cadherin and γ-catenin (Fig. 2c).

### Spire 1 knockdown perturbs Sertoli cell TJ-permeability barrier through changes in the distribution of proteins at the Sertoli cell–cell interface in vitro

**Fig. 3:**
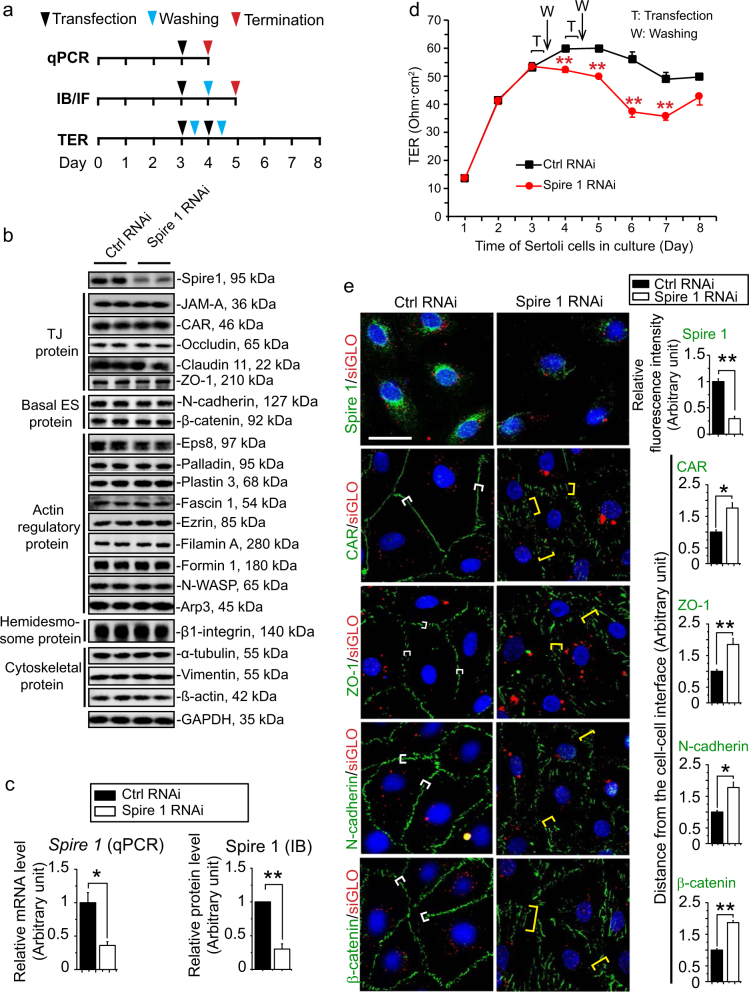
Knockdown of Spire 1 in Sertoli cell epithelium perturbs the TJ-permeability barrier function through disruptive distribution of TJ- and basal ES-proteins at the Sertoli cell–cell interface. **a** Illustration of the treatment regimen to obtain Sertoli cells for qPCR, IB or IF and TER analysis. **b** Sertoli cells harvested on day 5 were used to obtain cell lysates for IB (20 µg protein per lane) with corresponding specific antibodies listed in Table 1, illustrating Spire1 expression was considerably reduced. However, virtually all other BTB-associated proteins were not affected following Spire 1 knockdown, illustrating that there were no apparent off-target effects by using the Spire 1-specific siRNA duplexes vs. the non-targeting negative control siRNA duplexes. Glyceraldehyde-3-phosphate dehydrogenase (GAPDH) served as a protein loading control. **c** qPCR (left panel) and IB (right panel) was performed using RNAs or protein lysates obtained from Sertoli cells harvested on day 4 or 5, respectively, illustrating the expression of Spire 1 was reduced by ~70% when examined by qPCR or IB. Each bar in the histogram is a mean ± s.d. of *n* = 3 independent experiments. *P < 0.05; **P < 0.01 when compared to the corresponding control by Student’s *t*-test. **d** Sertoli cell Spire 1 knockdown perturbed the TJ-permeability barrier function. Each data point is a mean ± s.d. of quadruplicate bicameral units from a representative experiment. A total of *n* = 3 independent experiments were performed which yielded similar results. **P < 0.01 when compared to corresponding control by Student’s *t*-test. **e** IF analysis was used to confirm considerable decline in Spire 1 expression following RNAi based by immunofluorescence microscopy. Histogram on the right panel summarized the relative fluorescence intensity of Spire 1 in Spire 1 RNAi group vs. the corresponding control cells, illustrating at least a 70% knockdown. Sertoli cell *Spire1* knockdown also perturbed the localization and/or distribution of TJ proteins CAR (coxsackievirus and adenovirus receptor) and ZO-1 (zonula occludens-1), and basal ES proteins N-cadherin and β-catenin. In controls, TJ and basal ES proteins were tightly localized to the Sertoli cell–cell interface (see white brackets in control groups). After Spire 1 knockdown, these proteins were diffusively localized at the cell–cell interface (see yellow brackets in RNAi groups), moving into the cell cytosol. Co-transfection of siGLO Red Transfection Indicator (red fluorescence; Dharmacon/GE/Thermo Fisher) was used to illustrate successful transfection. Histograms in the right panel provide semi-quantitative analysis regarding changes in the relative distribution of fluorescence intensity at the cell–cell interface. Each bar is a mean ± s.d. of *n* = 3 independent experiments. *P < 0.05; **P < 0.01 when compared to corresponding control by Student’s *t*-test. Scale bar, 40 µm, which applies to other micrographs

Figure 3a is the regimen used to obtain lysates for immunoblot analysis shown in Fig. 3b. When Spire 1 was knockdown by approximately 70–75% based on IB (Fig. 3b, c) or qPCR (Fig. 3c), the steady-state levels of all the BTB-associated proteins including hemidesmosome protein were unaffected, illustrating the Spire 1 knockdown had no apparent off-target effects (Fig. 3b). However, Spire 1 knockdown was found to perturb the Sertoli cell TJ-permeability barrier function (Fig. 3d). When Sertoli cells from this experiment were examined by IF on day 5, a knockdown of Spire 1 by ~ 75% (based on fluorescence intensity analysis) was found to perturb the distribution of both TJ- (e.g., CAR, ZO-1) and basal ES- (e.g., N-cadherin, ß-catenin) at the Sertoli cell–cell interface (Fig. 3e). These proteins were internalized, possibly via endocytosis, since they no longer tightly localized at the cell cortical zone, but diffusively localized, re-distributing into the cell cytosol (Fig. 3e, see also histograms on the right panel).

### Knockdown of Spire 1 that perturbs TJ-barrier function is mediated by disruptive changes in Sertoli cell actin organization

**Fig. 4:**
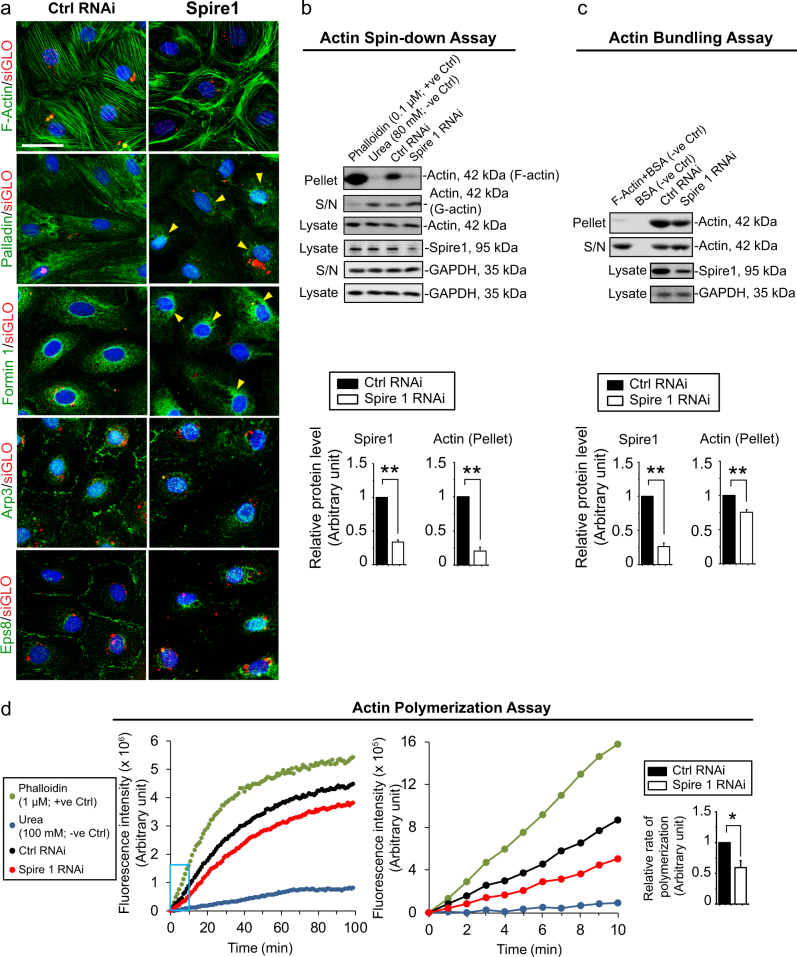
Knockdown of Spire 1 in Sertoli cells perturbs F-actin organization through disruptive changes in actin polymerization and bundling activity. **a** Knockdown of Spire 1 in Sertoli cells by RNAi was found to induce disruptive changes in the organization of actin microfilaments across Sertoli cell cytosol. These included truncation of actin filaments so that they no longer stretched across the cell cytosol as noted in control cells to support the Sertoli cell TJ-permeability barrier function as noted in Fig. 3d. These changes are supported by disruptive changes in spatial organization of actin bundling protein palladin and linear actin polymerization protein formin 1, which no longer stretched across the entire cell cytosol following Spire 1 knockdown as found in control cells, but retracted to localize closer to the cell nuclei (yellow arrowheads). Branched actin polymerization protein Arp3 and actin barbed-end capping and bundling protein Eps8 also no longer prominently expressed at the Sertoli cell–cell interface. Instead, Apr3 and Eps8 were internalized into cell cytosol, disrupting the ability to confer plasticity to the actin microfilaments near the cell cortical zone to support basal ES function to tighten the TJ-barrier. Co-transfection of rhodamine-siGLO indicator (red fluorescence) indicated successful transfection. Scale bar, 40 µm, which applies to other micrographs. **b** Actin spin-down assay was performed as detailed in Materials and Methods, which separated filamentous (F, in pellet) actin from globular (G, in supernatant (S/N)) actin in cell lysates from Sertoli cell cultures after Spire1 knockdown. As noted herein (bar graph in lower panel), Spire1 knockdown by ~ 70% reduced the level of F-actin in Sertoli cells considerably. GAPDH served as a protein loading control. Phalloidin (0.1 µM) and urea (80 mM) included in the Sertoli cell lysates for the assay served as the corresponding positive (+ve) and negative (−ve) control. Each bar in the histogram is a mean ± s.d. of *n* = 3 independent experiments. **P < 0.01. **c** Actin bundling assay was used to assess the ability of Sertoli cell lysates to bundle (pellet) premade F-actin in vitro vs. unbundled (i.e., individual) actin microfilaments (S/N). It was noted that Spire1 knockdown (see Sertoli cell lysates immunoblotted with anti-Spire 1 antibody) perturbed actin microfilament bundling activity in cell cytosol in Spire 1 RNAi group vs. control group (see also lower panel). GAPDH served as a protein loading control. In this assay, samples without Sertoli cell lysates but in the presence or absence of premade F-actin served as two corresponding negative controls. Each bar in the histogram is a mean ± s.d. of *n* = 3 independent experiments. **P < 0.01. **d** Actin polymerization assay was to assess the ability of Sertoli cell lysate to polymerize pyrene actin oligomers in vitro after *Spire1* knockdown. Kinetics of actin polymerization was assessed in 100 min (left panel) and data during the initial 10 min (middle panel, exponential filament growth phase, see the blue boxed area in left panel) of polymerization were used to assess the relative rate of polymerization and shown on the histogram on the right panel, illustrating *Spire1* knockdown perturbed actin microfilament polymerization kinetics.

Knockdown of actin nucleation protein Spire 1 by RNAi also found to perturb the organization of actin filaments across the Sertoli cell cytosol (Fig. 4a). For instance, in control Sertoli cells, actin filaments were stretched across the Sertoli cell cytosol in an organized fashion (Fig. 4a, top panel). However, considerably less actin filaments were found in Sertoli cell cytosol following Spire 1 knockdown; instead, actin filaments were retracted to be closer to cell nuclei (Fig. 4a). These changes were supported, at least in part, by changes in the distribution of other actin regulatory proteins. For instance, palladin (an actin bundling protein expressed by Sertoli cells^25^) no longer stretched across the Sertoli cell cytosol to support bundling of actin microfilaments to form the stress fibers following Spire 1 knockdown, but retracted to be closer to the cell nuclei (Fig. 4a). The distribution of formin 1, another actin nucleator, in Sertoli cells was also perturbed in which formin 1 retracted closer to the Sertoli cell nucleus. The distribution of Arp3 (branched actin nucleation protein) and Eps8 (actin barbed end capping and bundling protein) was also perturbed as these proteins no longer localized prominently at the Sertoli cell–cell interface to confer actin dynamics and/or plasticity but re-distributed into the cell cytosol, closer to the cell nuclei (Fig. 4a). Such disruptive changes in the distribution of these actin regulatory proteins thus impeded the organization of F-actin across the cell cytosol. Furthermore, following the knockdown of Spire 1, there were considerable reduction in the ratio of F:G ratio (Fig. 4b) and overall actin bundling activity (Fig. 4c). Furthermore, the kinetics and the rate of actin polymerization following Spire 1 knockdown were impeded (Fig. 4d).

### Knockdown of Spire 1 in Sertoli cells perturbs the organization of microtubule (MT)-based cytoskeleton

**Fig. 5:**
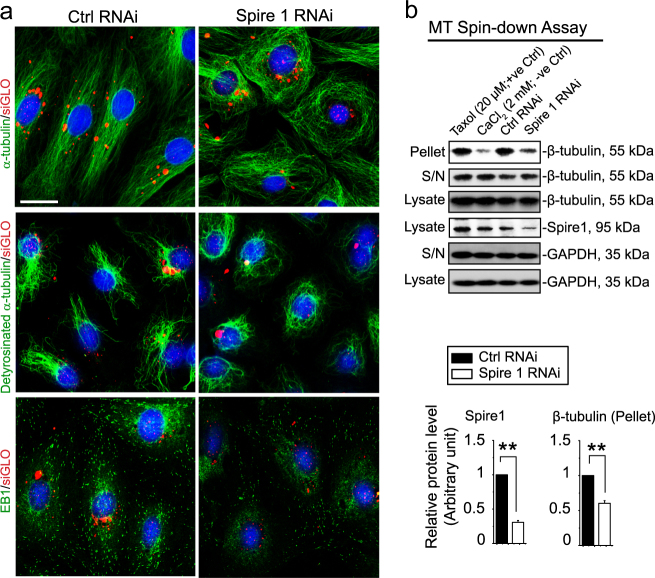
Knockdown of Spire 1 perturbs MT organization through disruptive changes in detyrosinated α-tubulin and EB1 distribution. **a** Knockdown of Spire 1 in Sertoli cells by RNAi perturbed the organization of MTs since α-tubulin (note: α- and β-tubulins are blocking blocks of MTs^12^) no longer stretched across the Sertoli cell cytosol as noted in control cells. Instead, MTs appeared to be largely truncated and wrapped around the cell nuclei, retracting from cell peripheries. On the other hand, detyrosinated α-tubulin (rendering MTs to become more stabilized) was found to wrap around the Sertoli cell nucleus loosely, displaying a pattern similar to α-tubulin following Spire 1 knockdown instead of stretching across the Sertoli cell cytosols as noted in control cells. Furthermore, the +TIP protein EB1 known to stabilize MTs and involved in promoting MT growth from the plus (+)-end also retracted from the Sertoli cell cytosol, and localized closer to the Sertoli cell nucleus following Spire 1 knockdown, dissimilar from control cells wherein EB1 scattered along the MTs that stretched across the long axis of the entire Sertoli cell. Co-transfection of rhodamine-siGLO indicator (red fluorescence) indicated successful transfection. Scale bar, 40 µm, which applies to other micrographs. **b** Microtubule spin-down assay was used to quantify the relative amount of polymerized microtubules (in pellet) vs. non-polymerized and free-tubulins (in supernatant) in Sertoli cell cytosol after knockdown of Spire 1. Knockdown of Spire 1, as confirmed by IB using Sertoli cell lysates, was found to perturb MT dynamics by reducing the level of polymerized MTs quantified in Sertoli cells. The presence of Taxol (20 µM) and CaCl_2_ (2 mM) in Sertoli cell lysates in this assay served as the corresponding positive (+ve) and negative (−ve) control, respectively. GAPDH served as the protein loading control. Each bar in the histogram is a mean ± s.d. of at *n* = 3 independent experiments. **P < 0.01

Spire 1 knockdown in Sertoli cells cultured in vitro was also found to perturb the organization of MTs across the cell cytosol (Figure 5a). α-Tubulins (note: α- and β-tubulins are the building blocks of MTs^12^) that constituted MTs, which stretched across the entire Sertoli cell cytosol to confer typical spindle-shape to these cells were retracted from cell peripheries but wrapped around the cell nuclei following Spire 1 knockdown (Fig. 5a). Besides α-tubulin, detyrosinated-α-tubulin, which rendered MTs to become stabilized (i.e., less dynamic) wherein its C-terminal Tyr (Y) was removed by exposing Glu (E) at its newly formed C-terminus^48–50^, also no longer stretched across the Sertoli cell cytosol as in control Sertoli cells, but wrapped and entangled around the cell nuclei following Spire 1 knockdown (Fig. 5a). Similar phenotype was also noted for EB1 (end binding protein 1, a plus (+) end tracking protein (+TIP) that binds to the plus (+) fast growing end of MTs to stabilize MTs) in which EB1 (seen as dot-like structures that laid across the +-end of MTs and stretched across the entire Sertoli cell cytosol) retracted from cell cytosol and localized closer to the cell nuclei (Fig. 5a). These findings were supported by a biochemical-based MT spin-down assay which monitored the relative level of polymerized MTs in Sertoli cells since Spire 1 knockdown led to a considerable reduction in polymerized MTs in Sertoli cells (Fig. 5b).

### Knockdown of Spire 1 in the testis perturbs spermatogenesis

**Fig. 6:**
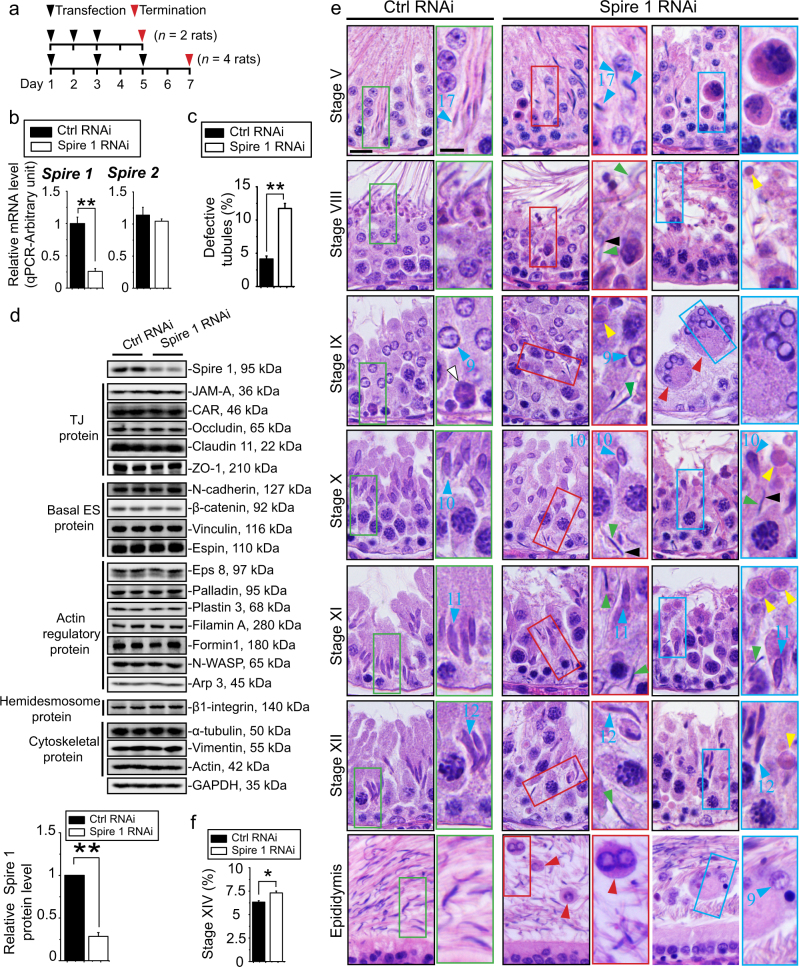
Knockdown of Spire 1 in adult rat testes in vivo perturbs spermatogenesis by disrupting the transport of spermatids and phagosomes across the seminiferous epithelium, and causing defects in spermatid polarity. **a** Illustration of the two regimens used to knockdown Spire 1 in the testis of adult rats in vivo with *n* = 2 rats or *n* = 4 rats in two independent experiments. Since the phenotypes obtained from the experiments of these two regimens were similar, these data were pooled for subsequent analysis. **b** The steady-state mRNA level of Spire 1 vs. Spire 2 in Spire 1 knockdown vs. non-target testes was analyzed by qPCR using GAPDH as an internal control. The steady-state mRNA level of *Spire1*, but not *Spire 2*, was reduced by ~70% following after Spire 1 RNA, illustrating the knockdown is specific to *Spire 1*. Each bar in the histogram is a mean ± s.d. of *n* = 4 rats. **P < 0.01. **c** Following Spire 1 knockdown by RNAi, a considerable number of seminiferous tubules displayed defects of spermatogenesis using the criteria outlined in Materials and Methods (and also shown in **d**) from *n* = 4 rats. In short, ~4% of tubules were found to have signs of defects in testes transfected with non-targeting negative control siRNA duplexes vs. 12% defective tubules, representing a 3-fold increase in defects in spermatogenesis following Spire 1 knockdown. Most of the defects (>90%) were found in stage V–XIV tubules. Each bar is a mean ± s.d. of *n* = 4 rats. About 5,000 tubules were randomly scored from each testis, and a total of 4 rats were scored. **P < 0.01. **d** Immunoblot analysis using lysates of testes supported Spire 1-specific knockdown without perturbing the expression of multiple BTB-associated proteins, categoried according to their functional roles, at the BTB, illustrating there was no apparent off-target effects following Spire 1 knockdown. GAPDH served as a protein loading control. Spire 1 was knockdown by at least ~70% based on data shown in the histogram in which each bar is a mean ± s.d. of *n* = 4 rats. **P < 0.01. **e** Histological analysis of cross-sections of testes at selected stages of the epithelial cycle and the epididymis following Spire 1 knockdown vs. controls illustrating: (i) the presence of giant multinucleated round spermatids (red arrowheads) which would undergo degeneration following Spire knockdown, (ii) failure in transport of phagosomes (yellow arrowheads) following Spire 1 RNAi vs. control testes (white arrowhead) when they should have been transported to the base of the epithelium for lysosomal degradation as found in corresponding control testes, (iii) failure in transport of and anchoring spermatids—the presence of step 19 spermatids (green arrowheads) embedded inside the epithelium in stage VIII tubules but also IX, X, XI, and XII tubules intermingled with corresponding step 9, 10, 11, and 12 spermatids (blue arrowheads) when they had already appeared, and numerous round (step 9) or multinucleated round spermatids, were persistently found in the epididymis following Spire 1 knockdown vs. control testes, and (iv) many elongated spermatids had defects of polarity by not orientating their heads toward, but deviated by approximately 90–180° from, the basement membrane (black arrowheads). Scale bar, 20 µm; inset, 10 µm. **f** A considerable increase in stage XIV tubules following Spire 1 was noted, possibly due to a reduction in the quantities of meiotic spindles to support meiosis, thereby delaying the completion of meiosis in stage XIV tubules

In vivo knockdown of Spire 1 in the testis was performed using Spire 1-specific siRNA duplexes vs. control siRNA duplexes using Polyplus in vivo-jetPEI as a transfection medium on day 1, 2 and 3 (i.e., a total of 3 transfections) (*n* = 2 rats) and terminated on day 5; or on day 1, 3 and 5 (also 3 transfections) (*n* = 4 rats) and terminated on day 7 (Fig. 6a). The two testes from the same animal received either Spire 1 siRNA duplexes or the control siRNA duplexes. Since the phenotypes from both sets of animals were similar, data were pooled for analysis and shown in Fig. 6. A study by qPCR using specific Spire 1 and Spire 2 primer pairs (Table 3) showed that only Spire 1 was knockdown by ~70%, but the expression of Spire 2 was unaffected (Fig. 6b), illustrating the knockdown was specific to Spire 1. In control testes, only ~4% of tubules had signs of defects in spermatogenesis, but following 70% knockdown of Spire 1 in the testis, about 12% of tubules, representing a 3-fold increase vs. controls, had defects in spermatogenesis using the criteria outlined in Materials and Methods based on histological analysis (Fig. 6c). IB analysis demonstrated that specific downregulation of Spire 1 in the testis by ~70% (Fig. 6d), which failed to induce changes in any of the BTB-associated protein, had no apparent off-target effects (Fig. 6d). Defects in spermatogenesis following Spire 1 knockdown most notably confined to stage VIII–XII tubules in which the transport of elongated spermatids and other intracellular organelles (e.g., phagosomes) across the epithelium were grossly disrupted (Fig. 6e). The most obvious defects detected in stages VIII–XII tubules were: (i) the presence of multinucleated round spermatids, (ii) persistent presence of step 19 spermatids in stages VIII–XII tubules embedded deep inside the epithelium which should have been released into the tubule lumen at stage VIII, (iii) step 19 spermatids trapped in the epithelium had defects in polarity with their heads pointed at least 90–180° away from the basement membrane, and (iv) persistent presence of phagosomes in the adluminal compartment near the tubule lumen when they should have been transported to the base of the epithelium for lysosomal degradation (Fig. 6e). These phenotypes were likely the result of defects in actin- and/or MT-mediated transport of spermatids and organelles (e.g., phagosomes). These changes also perturbed intracellular trafficking, causing the generation of giant multinucleated round spermatids (Fig. 6e), which would eventually undergo degeneration and be degraded by Sertoli cells. Furthermore, some round and multinucleated round spermatids were detected in the epididymis, not found in the control epididymis (Fig. 6e). Additionally, we also noted that there was a considerable increase in the percentage of stage XIV tubules during which meiosis took place because meiosis failed to undergo completion with the needed support of actin-based cytoskeleton that conferred the meiotic spindles (Fig. 6f).

### Knockdown of Spire 1 in the testis perturbs F-actin organization

**Fig. 7:**
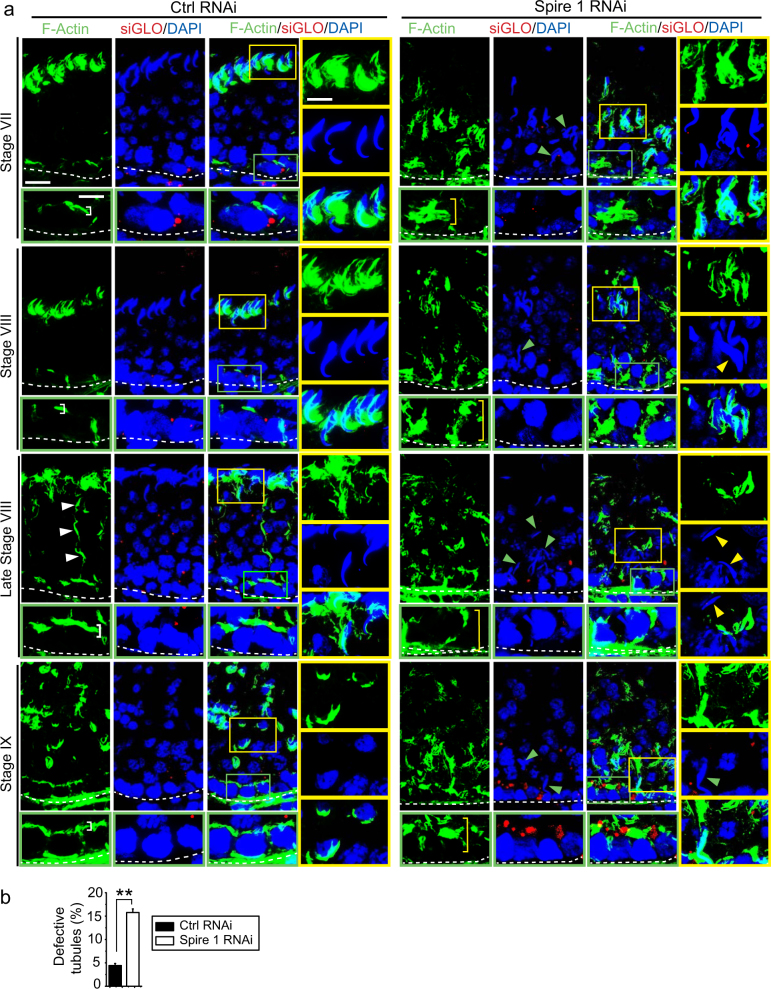
Knockdown of Spire1 in the testis in vivo perturbs F-actin organization in the seminiferous epithelium. **a** As noted in control testes transfected with non-targeting negative control siRNA duplexes, F-actin was prominently organized around the Sertoli–spermatid interface (yellow boxed areas) and also at the Sertoli cell–cell interface near the basement membrane (green boxed areas) to support the apical ES and basal ES/BTB function, respectively, as earlier reported^14, 73,74^, such as in stage VII, VIII, and IX tubules. Furthermore, F-actin also formed the track-like structures (annotated by white arrowheads), mostly notably detected in late stage VIII tubules, to support phagosome and spermatid transport. After Spire 1 knockdown, F-actin no longer tightly localized at the basal ES to support the BTB, instead, F-actin appeared as truncated/branched network, diffusely localized at the site (see yellow brackets in Spire 1 RNAi testes vs. white brackets in control testes) and no obvious actin-conferred track-like structures were noted. Also, F-actin that appeared as bulb-like structures prominently found at the concave side of spermatid heads in control testes were re-distributed, appeared as dis-organized structures by wrapping around spermatid heads after Spire 1 knockdown. Moreover, F-actin virtually was non-detected or considerably diminished in spermatids that had loss their polarity (i.e., no longer have the heads pointing toward the basement membrane, annotated by yellow arrowheads in Spire 1 RNAi group) or still embedded deep inside the seminiferous epithelium (annotated by green arrowheads) when they should have been released into the tubule lumen at spermiation. These defects of F-actin organization was detected even in tubules that appeared as ‘normal’ staged tubules following Spire 1 knockdown. Thus, when defects of F-actin organization was taken into consideration, the percentage of defective tubules were considerably higher (see **b** below). Co-transfection of rhodamine-siGLO indicator (red fluorescence) illustrated successful transfection in the tubules shown herein. Scale bar, 20 µm; inset, 10 µm. **b** When the % of defective tubules were scored based on disruptive organization of F-actin, the percentage of disorganized tubules was considerably increased as noted in this bar graph when compared to defective tubules noted based on histological analysis as noted in Fig. 6c. Each bar is a mean ± s.d. of *n* = 4 rats, ~2000 tubules were randomly scored from each testis to assess disruptive changes in F-actin organization in the Spire 1 knockdown vs. control group. **P < 0.01 by Student’s *t*-test

In control testes, F-actin was prominently detected at the apical ES and basal ES (Fig. 7a). At the apical ES, F-actin appeared as bulb-like structure, intensely localized at the concave (ventral) side of spermatid heads in stage VII and VIII tubules, but considerably diminished in late stage VIII tubules and expressed prominently again in stage IX tubules in step 9 spermatids at the front end of spermatid head surrounding the developing acrosome (Fig. 7a). Also noted are the track-like structures conferred by F-actin in late stage VIII tubules (see white arrowheads, Fig. 7a). F-actin was notably expressed at the BTB, located just above the basement membrane (annotated by a dashed white line) (Fig. 7a). However, following Spire 1 knockdown, there were gross disruption of F-actin networks in all stages of the tubules, as illustrated in stages VII–IX tubules shown in Fig. 7a. For instance, F-actin no longer appeared as bulb-like structure intensively localized at the concave side of spermatid heads but randomly expressed around the spermatid heads (Fig. 7a). Furthermore, step 19 spermatids remained trapped deep inside the epithelium in stage VII and VIII tubules, and had mis-organized F-actin wrapped around these spermatids, unlike the control testis wherein F-actin appeared as bulb-like structures localized at the concave side of spermatid heads (Fig. 7a). In late stage VIII and IX tubules, following Spire 1 knockdown, F-actin also grossly disorganized, dissimilar from control testes, (Fig. 7). For instance, no track-like structures conferred by F-actin were detected after Spire 1 knockdown. When changes in F-actin organization was used to assess the defective tubules, a considerable increase in defective tubules was noted following Spire 1 knockdown vs. the control group (Fig. 7b).

### Knockdown of Spire 1 in the testis perturbs protein distribution at the basal ES/BTB

**Fig. 8:**
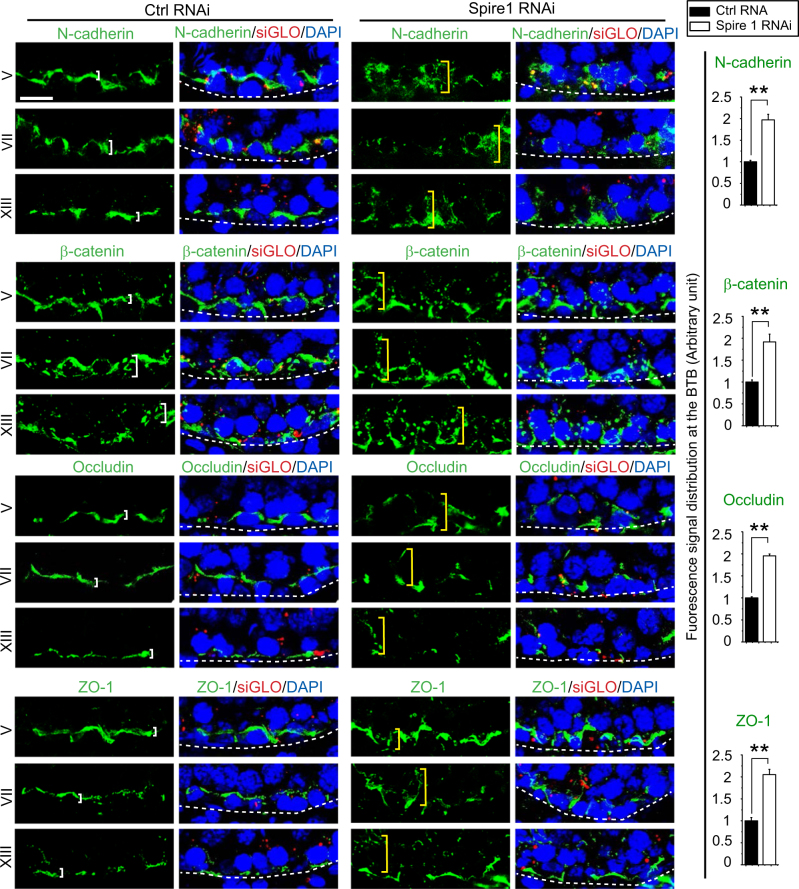
Knockdown of Spire 1 in the testis in vivo perturbs distribution of basal ES- and TJ-associated proteins that utilize F-actin for attachment at the BTB. In adult rat testes, basal TJ proteins N-cadherin and β-catenin, and TJ proteins occludin and ZO-1 are two of the constituent adhesion protein complexes that confer BTB its barrier function. As noted in control testes, these two adhesion protein complexes were tightly associated with the BTB (see white brackets) located above the basement membrane (annotated by the dashed white line) in selected staged tubules. However, following Spire 1 knockdown, these proteins were found to be diffusely localized at the BTB by extending considerably away from the site (see yellow brackets), well beyond the basement membrane (annotated by dashed white line). Co-transfection of rhodamine-siGLO indicator (red fluorescence) illustrated successful transfection. Histograms in the right panel summarized results of fluorescence images shown in the left panel regarding changes in the relative distribution of basal ES- or TJ-proteins in Spire 1 RNAi vs. control groups. Each bar in the histogram is a mean ± s.d. of *n* = 4 rats, and 50 randomly selected tubules of stage V–XIV were scored. **P < 0.01 by Student’s *t*-test. Scale bar, 20 µm

Following Spire 1 knockdown in the testis in vivoin vivo, considerable changes in the distribution of basal ES proteins N-cadherin and ß-catenin, and also TJ proteins occludin and ZO-1, were noted at the BTB, located above the basement membrane (annotated by the white dashed line) (Fig. 8). These basal ES and TJ proteins no longer tightly localized at the BTB as noted in control testes, instead, they were diffusely localized at the BTB (Fig. 8, see also histograms on the right panel), possibly due to disruptive changes in the organization of F-actin.

### Spire 1 knockdown perturbs F-actin organization in the testis through changes in the spatial expression of actin regulatory proteins Arp3 and Eps8

**Fig. 9:**
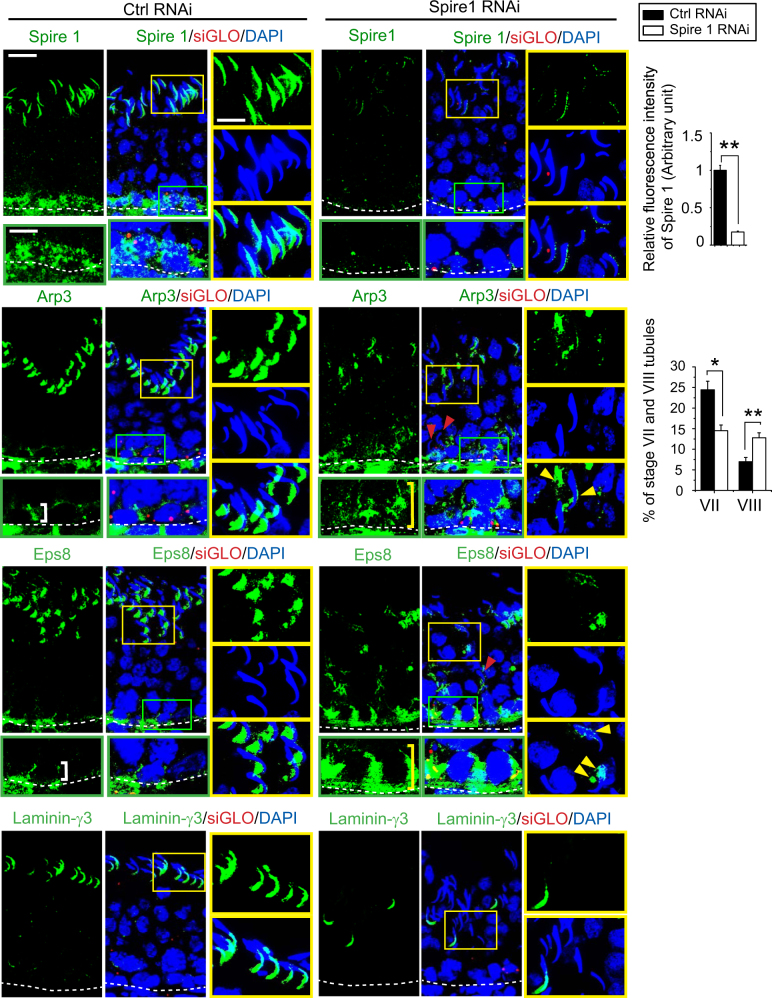
Knockdown of Spire 1 in the testis in vivo perturbs distribution of actin regulatory proteins at the apical and basal ES. The efficacy of Spire 1 knockdown was assessed by IF as Spire 1 fluorescence signal was considerably diminished following transfection of testes with Spire 1 siRNA duplexes vs. non-targeting negative control siRNA duplexes. The yellow and green boxed areas were magnified to illustrate disruptive changes at the apical and basal ES, respectively. In control testes, branched actin polymerization protein Arp3 and actin barbed-end capping and bundling protein Eps8 were robustly expressed as bulb-like structures at the apical ES on the concave side of spermatid heads in stage VII tubules. However, both proteins were considerably diminished; and in some instances, they were also mis-localized since they were found on the convex side or near the base of spermatid heads (see yellow arrowheads in yellow boxed areas in corresponding magnified images) following Spire 1 knockdown. Localization of these two actin regulatory proteins was also considerably perturbed at the basal ES since they no longer tightly associated with the BTB (white brackets) as noted in control testes, but diffusely expressed at the site (see yellow brackets) following Spire 1 knockdown. Changes in the distribution of these regulatory proteins at the apical ES as shown herein thus perturbed the spatial expression of an apical ES protein laminin-γ3 chain through its considerably downregulation and laminin-γ3 no longer prominently expressed at the tip of spermatid heads but considerably diminished following Spire 1 knockdown. Co-transfection of rhodamine-siGLO indicator (red fluorescence) illustrated successful transfection. Right panel summarizes results shown on the left panel, illustrating Spire 1 was silenced by close to ~80% based on IF analysis. There were also considerable changes in the frequency of stage VII and VIII tubules following Spire 1 knockdown vs. control testes due to defects in spermatid adhesion, making correct staging of tubules difficult. In control testes, about 24% and 7% of the total tubules were stage VII and VIII, respectively, consistent with earlier findings^51^. After Spire 1 knockdown, many stage VII tubules appeared as stage VIII tubules following Spire 1 knockdown, leading to a considerable decline or increase in the number of stage VII or VIII tubules scored in Spire 1 knockdown group as noted in bar graph on the lower right panel. Each bar in the histogram is a mean ± s.d. of *n* = 4 rats, and at least 200 stage VII or VIII tubules were randomly scored for comparison between the two groups. **P < 0.01, Student's *t*-test. Scale bar, 20 µm; inset, 10 µm

The efficacy of Spire 1 knockdown in the testis in vivo was confirmed by IF as noted in the top panel in Fig. 9a (see also histogram on the upper right panel). Changes in the organization of F-actin across the epithelium as noted in Fig. 7 were likely mediated through changes in the spatial expression of Arp3 and Eps8, which no longer robustly expressed at the concave side of the spermatid heads and restrictively expressed at the basal ES/BTB (Fig. 9). Instead, the expression of these two proteins were considerably diminished and also mis-localized compared to control testes (Fig. 9), thereby failing to support the preparation of elongated spermatids for their release at spermiation in late stage VIII tubules as well as their proper transport across the epithelium. This notion was supported by considerable reduction in the spatial expression of an apical ES protein laminin-γ3 chain at the spermatid heads (Fig. 9). The defects in F-actin organization thus induced premature release of elongated spermatids residing near the tubule lumen, but for those elongated spermatids that were near the base of the epithelium, they failed to be transported across the epithelium but remain trapped at the site (Fig. 9, red arrowheads). These changes that failed to support spermiation, altering the relative percentage of stage VII vs. VIII tubules, making it difficult to distinguish stage VII from putative stage VIII tubules due to premature spermiation, but also entrapment of step 19 spermatids in stage VIII tubules. For instance, in normal control testes (i.e., transfected with non-targeting negative control siRNA duplexes), VII and VIII tubules were found to be ~24% and ~7%, respectively, consistent findings of earlier reports^51,52^; but in Spire 1 knockdown testis, stage VII and VIII tubule were 15% and ~13%, respectively (Fig. 9, right panel).

### Spire 1 knockdown perturbs MT organization in the testis

**Fig. 10:**
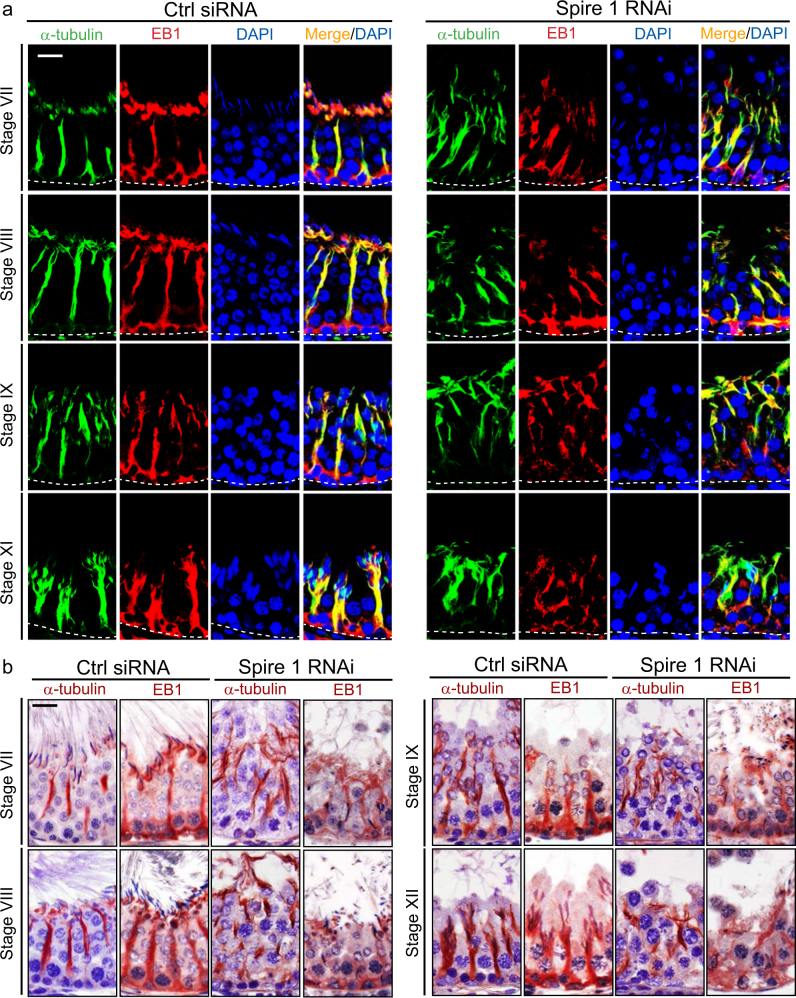
Knockdown of Spire 1 in the testis in vivo perturbs microtubule (MT) and EB1 organization or distribution in the seminiferous epithelium. IF (**a**) and IHC (**b**) were performed as described in Materials and Methods to assess if MT organization was perturbed following Spire 1 knockdown in the testis in vivo *vs.* control testes transfected with non-targeting negative control siRNA duplexes. In control testes, MTs and EB1 appeared as track-like structures that laid across the entire epithelium and aligned almost perpendicular to the basement membrane (annotated by dashed white lines). However, following Spire 1 knockdown, these MT-based tracks were either truncated or misaligned against the basement membrane. These disruptive changes were detected in virtually all staged tubules and representative findings in stage VII, VIII, IX, and XI tubules were shown herein. Scale bar, 20 µm which applies to corresponding images in the same panel.

Spire 1 knockdown in adult rat testes in vivo also perturbed the organization of MTs based on studies using IF (Fig. 10a) and IHC (Fig. 10b). MTs appeared as track-like structures that laid across the seminiferous epithelium and aligned perpendicular to the basement membrane (annotated by dashed white line) in all staged tubules as shown in control testes in representative stage VII, VIII, IX, and XI tubules (Fig. 10a, b). Following Spire 1 knockdown, MT organization was grossly disrupted. For instance, the MT-conferred tracks where grossly truncated, and for those MTs that remained appeared as considerably shortened tracks and were also misaligned since they no longer laid perpendicular to the basement membrane as in control testes (Fig. 10). Disrupted MT organization likely caused by the disrupted spatial expression of EB1, a known MT stabilizing +TIP protein^53^. For instance, EB1 was grossly truncated and misaligned in the epithelium after Spire 1 knockdown when visualized by IF (Fig. 10a) and IHC (Fig. 10b).

### Spire 1 knockdown perturbs the Sertoli cell BTB integrity in vivo

**Fig. 11:**
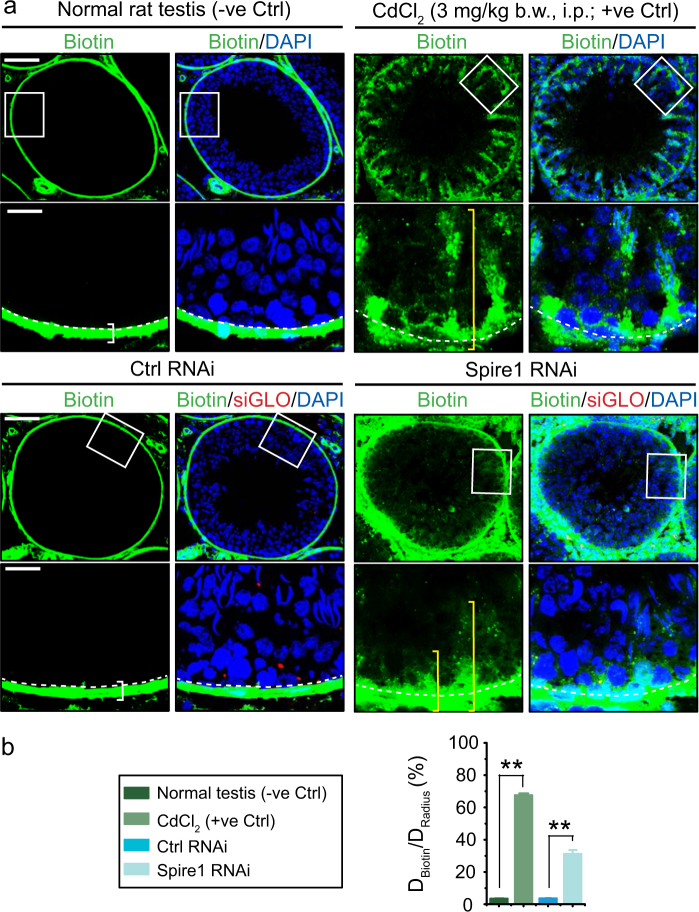
Knockdown of Spire 1 in the testis in vivo perturbs the Sertoli cell BTB. (**a**) In normal testes and testes transfected with non-targeting negative control siRNA duplexes (Ctrl RNAi), the BTB localized adjacent to the basement membrane (annotated by the white dash line) effectively blocked the diffusion of biotin (green fluorescence) from the basal (annotated by the white bracket in control groups) to the adluminal compartment. However, biotin freely diffused into the adluminal compartment in tubules from testes following treatment of rats with cadmium chloride (via i.m.) which is known to irreversibly disrupt the BTB function (positive control group) as annotated by the yellow bracket. Similarly, biotin was detected deep inside the adluminal compartment in testes following Spire 1 knockdown. Scale bar, 350 µm, first and third panel; 80 µm in second and fourth panel. The white boxed area was magnified and shown in the lower panel. **b** Semi-quantitative data illustrating the BTB was grossly disrupted by cadmium chloride, but a knockdown of Spire 1 RNAi also perturbed the BTB integrity considerably. Each bar is a mean ± s.d. of *n* = 3 rats. **P < 0.01 by Student’s *t*-test

Findings shown in Figs. 7–10 support the notion that a knockdown of Spire 1 would perturb the BTB in vivo, we thus performed the BTB integrity assay to examine this possibility. As shown in Fig. 11, a knockdown of Spire 1 indeed perturbed the Sertoli cell BTB function in vivo (Fig. 11a), and this disruptive effect is statistically significant as shown in Fig. 11b.

## Discussion

Spire is as an actin nucleator known to generating long stretches of linear actin microfilaments efficiently to support epithelial and endothelial cell function^4,22,54–56^. Spire either works alone by interacting with the barbed end of the actin filament^57^ or it works in concert with formin to create the Spir/formin actin nucleator complex^2,58,59^ to induce actin polymerization. The actin polymerization activity in Spire is conferred by the central repeat of four WH2 (WASP-homology 2) domains containing the consensus sequence of LKKV commonly found in most actin filament nucleators, such as Spire 1^4,58,60,61^. Thus, it is not surprising to see that Spire 1 is robustly expressed in the apical ES and basal ES ultrastructures since these are the unique actin-rich adherens junctions in the testis. Furthermore, the ES is supported by an extensive network of actin microfilament bundles that lay adjacent to the MT network, both of which undergo extensive remodeling, namely disassembly (depolymerization or cleavage) and reassembly (polymerization/nucleation) to confer plasticity to the ES to support germ cell transport and endocytic vesicle-mediated intracellular protein trafficking^12,14,62,63^. In short, apical and basal ES are dynamic AJ structures that require continuous remodeling to support the transport of spermatids and preleptotene spermatocytes across the adluminal compartment and the BTB, respectively, illustrating actin microfilaments that are assembled in bundles to support ES function undergo rapid re-organization. On the other hand, Spire 1 is co-localized, virtually superimposable, with apical ES constituent proteins such as β1-integrin^45^, nectin 2^46^ and nectin 3^46^ at the convex side of spermatid heads; and also basal ES proteins N-cadherin^64^ and γ-catenin at the BTB. These ES proteins are known to utilize actin microfilaments for their attachments^65^. In short, the precise co-localization of Spire 1 with these apical and basal ES cell adhesion protein complexes as noted herein is necessary to confer actin and possibly MT dynamics to support spermatogenesis. Thus, it is not unexpected that a knockdown of Spire 1 in Sertoli cells in vitro or in the testis in vivo was found to perturb the organization of F-actin network, impeding spermatid adhesion function, leading to their premature exfoliation from the seminiferous epithelium, but also perturbing spermatid transport across the epithelium. Also, some step 19 spermatids remained trapped deep inside the epithelium in stages IX, X, XI, and even XII tubules when they should have been released into the tubule lumen at stage VIII of the epithelial cycle. This is likely due to the loss of track-like structure conferred by actin filaments, and also the loss of tracks conferred by MTs, following Spire 1 knockdown, which are known to support cellular transport in other epithelia^11,12,66,67^. At present, it is not known if Spire 1 is working in concert with formin 1 as a Spire 1/formin 1 nucleation complex in the testis to support spermatogenesis. On the other hand, studies have also shown that Spire 1 is working with small GTPase Rab11 and actin-specific motor protein myosin Vb [2] as well as Rab 3A to modulate actin dynamics^68^. The possibility that Spire 1 is interacting with these other possible partners to mediate actin nucleation in the seminiferous epithelium during the epithelial cycle will require further investigations. Also, it is not known if the phenotypes noted herein after knockdown of Spire 1 were due to a disorganization of either actin, MT or both since the organization of both cytoskeletons was perturbed. However, it is likely that the actin-based cytoskeleton is the primary target of Spire 1 knockdown because Spire 1 is an actin nucleator. The primary disruption of actin organization thus impedes intracellular protein trafficking that causes a secondary disruption of MT organization. This possibility, however, must be carefully evaluated in future studies.

In this context, it is of interest to note that our findings that the mRNA of *Spire 1* and *Spire 2* genes are highly expressed in the testis are consistent with earlier reports^69,70^. Spire 1 and Spire 2 proteins were also shown to interact with formin 2 to facilitate proper alignment of metaphase spindle to support oocyte maturation during meiosis I in oocytes^69^. Interestingly, formin 2 deficient female mice (*Fmn2*^−/−^ female mice) were sub-fertile, having recurrent pregnancy loss due to polyploid embryo formation^71^. However, both *Spire 1* and *Spire 2* genes had to be knocked down to generate the *Fmn2*^−/−^ phenotype in oocytes^69^, suggesting Spire 1 and Spire 2 may have some redundant function. As such, it is not surprising to note that *Spire 1* KO mice using a gene trap approach to obtain the mutant mice, both male and female mice were fertile^72^. One would argue if Spire 2 could supersede the lost function of Spire 1, why would a transient knockdown of Spire 1 lead to the phenotypes detected in the testis as reported herein? This is likely that while both Spire 1 and Spire 2 are present in the testis to support spermatogenesis, a sudden and unexpected loss of Spire 1 that led to the noted phenotypes—defects in spermatid and phagosome transport and adhesion, as well as loss of spermatid polarity due to a disorganization of actin- and MT-based cytoskeletons—would take time for the Spire 2 to kick-in the lost function of Spire 1, thereby revealing the significance of Spire 1 to support spermatogenesis. This conclusion is supported by the finding that the steady-state mRNA level of Spire 2 in the Spire 1 knockdown testis was unaffected. Furthermore, both in vitro and in vivo findings are consistent with each other that a knockdown of Spire 1 would impede both F-actin and MT organization in Sertoli cells in the testis. Furthermore, the phenotypes detected in the testis following Spire 1 knockdown also mimic those detected in an earlier report when formin 1 was silenced by ~70% in the testis^20,21^, supporting the notion that Spire1/formin 1 may be a crucial actin nucleator complex to support spermatogenesis.

It was unexpected that a knockdown of Spire 1 in Sertoli cells cultured in vitro or in the testis in vivo perturbed MT organization. It is possible that the disruptive effects of Spire 1 on MT organization was the result of a transient loss of Spire 1 actin nucleation activity following its knockdown which impeded F-actin-dependent intracellular protein trafficking. This thus caused a disruptive spatial expression of MT regulatory proteins, such as EB1 and/or MARKs, which in turn could perturb MT organization, leading to disruptive changes in MT organization. These possibilities must be carefully evaluated in future studies.
